# A Case Report of Pulsed Radiofrequency Plus Suboccipital Injection of the Greater Occipital Nerve: An Easier Target for Treatment of Cluster Headache

**DOI:** 10.3389/fneur.2021.724746

**Published:** 2021-09-28

**Authors:** Enrico Belgrado, Andrea Surcinelli, Gian Luigi Gigli, Gaia Pellitteri, Chiara Dalla Torre, Mariarosaria Valente

**Affiliations:** ^1^Neurology Unit, Department of Neuroscience, Udine University Hospital, Udine, Italy; ^2^Clinical Neurology Unit, Department of Neuroscience, Udine University Hospital, Udine, Italy; ^3^Department of Medical Area (DAME), University of Udine, Udine, Italy

**Keywords:** greater occipital nerve, percutaneous pulsed radiofrequency, suboccipital steroid injection, treatment resistant cluster headache, prophylactic therapy

## Abstract

**Introduction:** In cluster headache, the efficacy of suboccipital steroid injection is notable within a few days, although few data are available about the duration of efficacy. A combination treatment, consisting of suboccipital steroid injection plus pulsed radiofrequency, could potentially lead to long-term benefit. Evidence about pulsed radiofrequency of the greater occipital nerve is lacking.

**Patients and Methods:** We retrospectively describe a series of four cluster headache patients treated with suboccipital steroid injection plus pulsed radiofrequency of the greater occipital nerve.

**Results:** All patients achieved a 50% reduction in attack frequency in the 7 days after the first treatment. Moreover, a long pain-free remission period up to 15 months was noted.

**Conclusion:** Suboccipital steroid injection plus pulsed radiofrequency of the greater occipital nerve might have both acute and prophylactic effects in cluster headache. The greater occipital nerve is more accessible to pulsed radiofrequency than other targets.

## Introduction

Suboccipital Steroid Injection (SSI) is a strongly recommended prophylactic therapy for Cluster Headache (CH), according to the American Headache Society Guidelines (1). In clinical practice, SSI has been frequently accompanied by the injection of local anesthetics, especially before the publication of the AHS guidelines. The efficacy of SSI is notable within a few days, potentially due to the local effect of steroids (2). As suggested in a nerve injury model, steroids block neurotransmission in normal C fibers and decrease heat and mechanical hyperalgesia (3). A systemic effect has also been suggested, although important differences between oral steroids and SSI have been highlighted in previous papers. Differently from SSI, high dosages of oral steroids are usually required to reduce attacks and the same attacks tend to resume when oral steroids are tapered down (2, 4).

Considering the only two double-blind, placebo-controlled studies evaluating SSI in CH, a single injection using a mixture of xylocaine and betamethasone was used by Ambrosini et al. (5), while up to three injections with cortivazol alone were performed by Leroux et al. (2). The efficacy of SSI has also been suggested by open label studies using a single injection of lidocaine and methylprednisolone (4), or lidocaine and betamethasone (6). In our clinic, up to three injections of bupivacaine and triamcinolone are routinely utilized for the transitional treatment of CH.

Data regarding the long-term efficacy, safety, and tolerability of SSI in CH are scarce. In both of the abovementioned double-blind, placebo-controlled studies, more than 50% of treated patients reached a pain-free remission period up to 1 month after treatment (61 and 52%, respectively) (2, 5). Afridi et al., in the CH group (19 patients), reported a mean duration of complete response (pain free) and of partial response (reduction in severity or frequency of headache by >30%) of 17 and 52 days, respectively. Interestingly, Ambrosini et al., reported a long pain free remission period up to 6 months in 38% of treated patients, although they were all episodic CH (ECH) patients with a relatively lower frequency of attacks at baseline (1–2 daily attacks).

An interesting review and meta-analysis about efficacy and safety of SI for the treatment of CH (7) reported poor quality of evidence for this type of therapy, in line with other preventive treatment options for CH. Authors summarize and suggest SI for ECH patients as the main preventive option. In ECH patients with long lasting bouts, SI could be offered alongside an oral preventive treatment to ensure a steady attacks control. In CCH patients, quarterly SI can be effective.

Although less used, another apparently safe and effective treatment for patients with CH Pulsed Radiofrequency (PRF). This neuromodulation technique, through rapidly changing electric fields, causes an intense repolarization of the excitatory C fibers with a subsequent inhibition of pain signal transmission (8). PRF could also modulate pain regulatory gene expression along the nociceptive pathway (9), potentially leading to a more long-term benefit.

On this basis, a combined treatment of SSI plus PRF has been used in our clinic as a transitional therapy for drug-resistant CH, for CH patients with a narrow range of therapeutic opportunities, and for ECH with long lasting bouts.

Sphenopalatine ganglion (SPG) (10), C1–C2 nerve roots (11), and C2 nerve roots (12, 13) have been investigated as PRF targets in CH. PRF of the greater occipital nerve (GON) has been preferred in our clinic for the treatment of CH since, unlike the other PRF targets, it is less invasive, not requiring a computed tomography (CT), is easier to perform, and potentially associated with fewer adverse events. Moreover, since GON arises from the dorsal primary ramus of the second cervical nerve with a contribution from the third cervical nerve, it represents an important share of afferent fibers to trigeminocervical complex (TCC). As known, TCC plays a pivotal rule in the pathophysiology of CH.

We are not aware of other studies reporting effects of SSI plus PRF of the GON. Here we retrospectively review our series of CH patients who underwent SSI +PRF in the region of the GON.

## Materials and Methods

Between March 2012 and November 2020, 29 subjects were evaluated and treated because of CH or probable CH at the Headache Center of Clinical Neurology Unit of Udine. It is a tertiary level center providing advanced imaging techniques (MRI, PET, EEG, and TCD) 5 days per week for outpatient visits, a broad range of therapeutic opportunities (monoclonal antibodies, botulinum toxin, anesthetic and/or steroid local injections, and ketogenic diet), and the possibility for hospital admission. Patient assessment and treatment planning were made by a neurologist who specialized in headaches. In accordance with the local legislation and institutional requirements, ethical review and approval was not required for the study. All participants gave their written informed consent for treatment after receiving full oral information. They also signed the consent form for retrospective data collection and for the publication of the manuscript, including any accompanying images.

The following were the inclusion criteria: patients with CH; CH that failed to respond to at least three agents with positive evidence (level of evidence A, B, or C) in accordance with the American Headache Society Evidence-Based Guidelines (1); age ≥ 18 years; signed inform consent for the procedure; and signed consent form for the retrospective collection of data.

Exclusion criteria were as follows: age < 18 years; cognitive impairment; or responder CH patients (first, second, or third line therapy).

The average frequency of attacks was collected in the last 2 weeks before treatment (T0), in the 7 days following treatment (T1), and in the last 2 weeks of the third month following treatment (T2). Responders were defined by 50% reduction in attack frequency; complete responders were defined by disappearance of the attacks.

**Figure 1:**
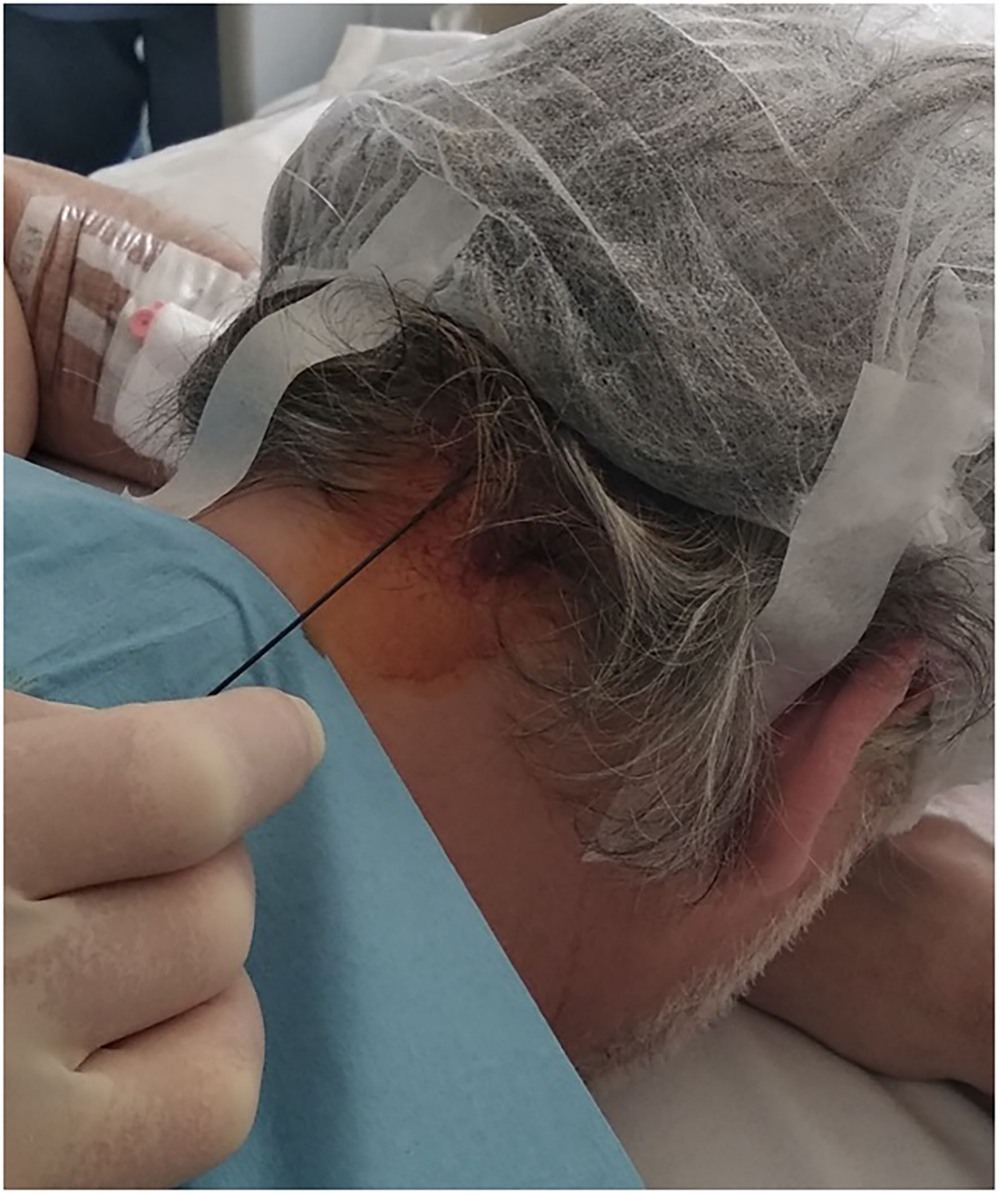
Pulsed Radiofrequency of the GON.

All PRF treatments were performed using a portable ultrasound system with a 2–50 Hz multifrequency transducer (Cosman G4™ RF Generator device). Patients were asked to lie prone on the table with the head flexed forward. To locate the nerve, we searched for the occipital artery in the medial one-third of the line between inion and mastoid process. GON is usually located just medial to the occipital artery. The scalp was then cleaned with betadine. A 22-gauge needle was advanced using this landmark-based technique shown in Figure 1. A selective sensory stimulation (50 Hz) showed concordant pain or paresthesia along skin territory innervated by the GON (the back of the scalp up to the vertex) below 0.5 V. This sensory stimulation confirmed the correct position of the electrode. PRF treatment consisted of two cycles of 180 s each, with a pulse duration of 20 ms at 2 Hz, and a 60-s interval between each cycle. The selective sensory stimulation was performed before every treatment. To prevent tissue damage, temperature was maintained below 42°C. The treatment was always performed ipsilateral to the pain.

Then, a single injection of steroids and local anesthetic (bupivacaine 5 mg plus triamcinolone 20 mg) was performed in the same spot of PRF treatment.

## Results

Among 29 patients, 27 were diagnosed with CH while two patients were diagnosed with probable CH, according to the International Classification of Headache Disorders (ICHD-3; ICHD-3 beta; ICHD-2) (14–16).

Due to a quite large period covered by the study, the patients were diagnosed in accordance with different International Classification of Headache Disorders. In total, four male patients were selected and included in this series. Specifically, the first two patients were diagnosed with CCH and ECH respectively in accordance with ICHD-3 beta; the last two patients were diagnosed with ECH in accordance to ICHD-3.

**Figure 2:**
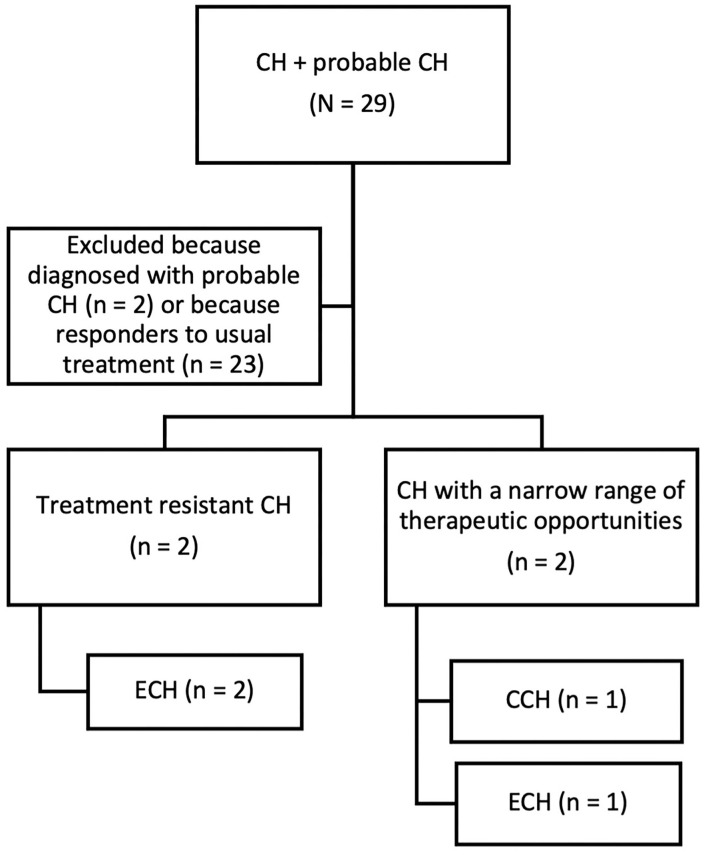
Flow chart of retrospective patients' selection. CH, Cluster Headache; CCH, Chronic Cluster Headache; ECH, Episodic Cluster Headache.

Furthermore, those four male patients were diagnosed with treatment resistant CH (two patients) and CH with a narrow range of therapeutic opportunities for comorbidities (two patients) as shown in Figure 2. As regards the latter category of patients, lithium was contraindicated in one patient due to psoriasis and verapamil was contraindicated in another patient due to left ventricular failure.

Although the current ICHD-3 does not include a definition of refractoriness for CH, and nor did the previous ICHD-3 beta, considering the consensus statement on clinical definition of refractory CCH from the European Headache Federation (EHF) (17), we define treatment resistant CH as a headache fulfilling ICHD-3 criteria (or previously ICHD-3 beta) for CCH, who failed to respond to at least three agents that showed efficacy over placebo in randomized controlled studies, used at the maximum tolerated dose over a sufficient period of time. Although the abovementioned EHF consensus does not take into account the definition of refractory ECH, the patients with this diagnosis were defined as refractory based on the failure of three agents with positive evidence (level of evidence A, B, or C) in accordance with the American Headache Society Evidence-Based Guidelines (1).

The mean age at onset was 36 years (range, 16–52 years). Mean duration of headache history was 25 years (range, 3–36 years). Episodic cluster headache (ECH) was noted in three patients and chronic cluster headache (CCH) in one patient. Pain was reported as orbital and/or supra-orbital in all patients. Moreover, one patient experienced pain side shift between clusters. Also in this patient, the treatment was performed unilaterally, always ipsilaterally to the pain's side (in total three treatments were administered, two on the right, and one on the left).

Before the first treatment, the average frequency of attacks was six per day (range, 2–8 attacks). Regarding prophylaxis, all patients had been previously treated with SSI. The response to SSI treatment was transient for all patients (10–16 days), complete in only two of them, and incomplete in the other two. The average of previous SSI treatments was seven (range, 3–11). The minimum latency between last SI and the combined treatment was 1 month (range, 1–4; median 2).

Other demographic data and headache characteristics are summarized in Table 1.

**Table 1:** Demographic data, headache characteristics.

	**Patient 1**	**Patient 2**	**Patient 3**	**Patient 4**
Age	55	55	52	82
Sex	Male	Male	Male	Male
Type of CH	CCH	ECH	ECH	ECH
Age at onset of CH	27	52	16	50
Location of pain	Alternating sides between clusters (orbital)	Right (orbital and supra-orbital)	Right (orbital and supra-orbital)	Right (orbital and supra-orbital)
Associated autonomic manifestations and/or agitation/restlessness	Nasal congestion, miosis, ptosis, lacrimation, agitation	Nasal congestion, lacrimation, agitation	Nasal congestion, miosis, ptosis lacrimation, agitation	Nasal congestion, miosis, ptosis lacrimation, agitation
Attacks/day at T0	5 (1st treatment); 4 (2nd treatment); 5 (3rd treatment)	2 (1st treatment); 2 (2nd treatment);	7 (1st treatment)	8 (1st treatment)
Previous prophylaxis	SI Verapamil Prednisone Topiramate Sodium valproate	SI Verapamil Lithium	SI Verapamil Lithium Flunarizine Prednisone	SI Verapamil
Number of previous SI	11 (6 right; 5 left)	8	4	3
Ongoing prophylaxis	Verapamil 480 mg	Verapamil 360 mg	None	None

*CH, Cluster Headache; CCH, Chronic Cluster Headache; ECH, Episodic Cluster Headache; GON Greater Occipital Nerve; Attacks/day at T0, Average daily attacks in the last two weeks before the first PRF treatment; N.A., Not Applicable/Not Available; SI, Suboccipital Injection*.

All patients achieved a 50% reduction in attack frequency (responders) in the 7 days following the first treatment (T1). Interestingly, two of them became complete responders at T2, starting from a partial response at T1. The three patients diagnosed with ECH achieved a complete resolution of the ongoing cluster (complete responders) after one to two treatments at T2, whereas the patient diagnosed with CCH achieved a complete resolution after three treatments at T2.

All patients achieved a long pain-free remission period. Until today, two patients are still free of attacks (pain free remission period up to 15 and 6 months, respectively) whereas two patients recurred after 15 and 3.5 months respectively.

A summary of results is reported in Table 2. No serious adverse events were noted. Nearly all patients complained with mild local discomfort during PRF, mainly due to heat and a reported sensation of tingling, buzzing, or vibrating.

**Table 2:** Overview of results.

		**Patient 1**	**Patient 2**	**Patient 3**	**Patient 4**
1st treatment	Outcome	Responder (T1) Responder (T2)	Responder (T1) Responder (T2)	Responder (T1) Complete responder (T2)	Responder (T1) Complete responder (T2)
2nd treatment	Outcome	Responder (T1) Responder (T2)	Complete responder (T1) Complete responder (T2)		
3rd treatment	Outcome	Complete responder (T1) Complete responder (T2)			
Pain-free remission period		15 months	3.5 months	Ongoing (15 months)	Ongoing (6 months)

*PRF+SI, Percutaneous pulsed radiofrequency plus suboccipital injection; T1, average daily attacks in the 7 days following treatment; T2, average daily attacks in the last 2 weeks of the 3rd month following treatment (T2); Outcome: non-responder (<50% reduction), responder (≥50% reduction), complete responder (100% reduction)*.

## Discussion

A combination treatment, combining SSI and PRF of the GON, in accordance with our data, might have both acute and prophylactic effects in CH patients. Despite the preliminary nature of our data, there are indeed some interesting aspects that need to be underlined.

All patients achieved, at least, a 50% reduction in attack frequency in the 7 days following the first treatment (T1), in accordance with the prompt efficacy of SSI, notable within a few days.

In contrast to SSI alone, remission persisted up to 3 months in all patients. Moreover, two responders at T1 became complete responders at T2 after the first treatment alone, possibly suggesting a synergic effect of SSI+PRF. After two or three cycles, also in the other CH patients, we observed a disappearance of the attacks (complete responders). In addition, free remission period persisted up to 15 months, potentially due to a long-term benefit of SSI+PRF. As mentioned above, PRF could modulate pain regulatory gene expression along the nociceptive pathway, in order to achieve a longer pain-free remission period. On the other hand, due to the episodic nature of CH in three patients, as they all suffered from long lasting bouts, we cannot exclude a resolution of the ongoing cluster related to the natural course of the disease.

Interestingly, although the treatment was performed targeted to the GON, all patients reported orbital and/or supraorbital pain relief. As mentioned before, GON represents an important share of afferent fibers to TCC.

Further, all patients were affected by a drug-resistance form or other first and/or second line prophylaxis were contraindicated due to comorbidities, suggesting a potential rule of SSI+PRF also in this difficult category of patients.

On the other hand, our study has several limitations. First of all, the retrospective nature of the study could potentially lead to patient selection bias. Second, the sample is quite small. Third, the absence of a control group cannot exclude a placebo effect. Fourth, two definite CH patients were on oral prophylaxis and, although drug posology was maintained as stable through the study period, we cannot exclude a possible interference. Although patients were all previously treated with SI alone with at least 1 month's interval before the combined treatment, we cannot exclude a possible cumulative effect of steroids. Lastly, the long pain-free remission period observed in some patients could also be related to the natural resolution of the ongoing cluster.

In conclusion, SSI+PRF, performed in the region of the GON, might be a reasonable approach in patients who suffered from CH, particularly in the drug-resistant form and/or when other first and second line treatments are contraindicated. Moreover, it could be a useful approach in patients who suffered from ECH with long lasting bouts (usually longer than 1 month) in order to achieve pain relief within a few days possibly without starting alongside an oral steroid to ensure a steady attack control. Compared to other sites of application of PRF, in our experience the choice of GON for treatment is easier to perform and has the advantages of being less invasive and of not requiring a CT.

Further studies are needed to confirm our preliminary data of efficacy and tolerability.

## Data Availability Statement

The original contributions presented in the study are included in the article/supplementary material, further inquiries can be directed to the corresponding author/s.

## Ethics Statement

Ethical review and approval was not required for the study on human participants in accordance with the local legislation and institutional requirements. Written informed consent for participation was not required for this study in accordance with the national legislation and the institutional requirements.

## Author Contributions

EB and AS: design and conceptualized study, acquisition of data, analyzed the data, and drafted the manuscript for intellectual content. GG and MV: design and conceptualized study and revised the manuscript for intellectual content. CD: acquisition of data and revised the manuscript for intellectual content. EB, AS, GG, and MV: design and conceptualized the study. EB, AS, and CD were involved in data acquisition. GG, GP, CD, and MV revised the manuscript for intellectual content. All authors contributed to the article and approved the submitted version.

## Conflict of Interest

The authors declare that the research was conducted in the absence of any commercial or financial relationships that could be construed as a potential conflict of interest.

## Publisher's Note

All claims expressed in this article are solely those of the authors and do not necessarily represent those of their affiliated organizations, or those of the publisher, the editors and the reviewers. Any product that may be evaluated in this article, or claim that may be made by its manufacturer, is not guaranteed or endorsed by the publisher.
